# The System of Living-In

**Published:** 1920-12-04

**Authors:** 


### THE MATRONS' AND SISTERS' DEPARTMENT.

### THE SYSTEM OF LIVING-IN.

### Its Suggested Abolition for the Trained Staff.

A Matron writes:

There is no problem more perplexing in the nursing world to-day than the satisfactory housing of the institutional staff. The cost of building is prohibitive, and even if the funds are available many hospitals cannot secure a suitable site within a reasonable distance; so the difficulty of transport during inclement weather presents itself. The working capacity of a probationer will not be increased if she reports on duty after a struggle with wind and rain.

When the Hours of Employment Bill becomes law the present difficulty will be aggravated. Accommodation is not elastic; to fit three bedsteads into space which is inadequate for two is a practical impossibility. Hospital committees must therefore either find a definite solution or reduce the number of beds available for patients for an indefinite period. The housing of the nursing staff is a matter which reacts directly upon the welfare of the sick; it is therefore a matter for national consideration. It cannot be brushed aside as a matter of little moment. If the beds of public institutions supported by voluntary contributions are to be closed down the nation will have the right to ask for a clear and lucid explanation.

Where must we seek for the solution of this problem? The Minister of Health (Dr. Addison) has definitely refused to sanction the "living-out system" for probationers, and his decision is upheld by many responsible matrons of wide experience and liberal views. Probationers are students "in training"; they are subject to severe discipline. They have to be educated in responsibility, especially they have to be taught that any abuse of privilege or liberty affects not only their own well-being, but that of others, since lack of efficiency on their part reacts on the patients entrusted to their care.

But when their training is at an end, when after three or four years of firm discipline, when their nursing education with all that it entails is complete, when they are fully trained, responsible professional women, why should they not be permitted to "live out"?

To-day we are losing from our hospitals sisters and staff nurses whose services it would be a pleasure and a profit to retain, because they can no longer endure the monotonous, devitalising effect of institutional life. As a nation we are renowned for our love of "home." It is a quality inherent in the English race. Is it necessary, is it wise, to insist that women who are particularly fitted for institutional work must sever themselves from their kin? In by-gone days, owing to the long hours on duty, it was imperative that sisters and staff nurses should live in the institutions in which they worked, but now that the eight-hour day is being adopted surely this necessity no longer exists.

In our hospitals to-day we need women of broad sympathies, women who can view life from a national standpoint, women who are in touch with a wide circle and who realise the interdependence of each stratum of social life. These are the women who are leaving our hospitals as soon as their training is complete. Year after year of institutional life tends to introspection. Slowly but surely sisters and nurses drop their outside interests and lose touch with their friends; their hospital becomes an octopus whose tentacles are drawing them away from legitimate recreation into an unnatural state of mental and physical indifference. Can we afford, as a nation, to allow women who are peculiarly fitted for hospital work to drift into other branches of the Services because they are not prepared to segregate themselves during their economic life in an institution?

There are many women to-day who would willingly enter our nurse-training schools if they were assured that at the completion of their training they could lead the ordinary normal life of their sisters and friends.

Surely this solution of a most difficult problem is worthy of a trial. We must find a way out of the present impasse, and a way which will benefit all sections of the community.

The question of expense presents itself, but if we take into consideration the cost of the building, equipment, and upkeep of residential quarters, with the domestic service entailed, it is very doubtful if allowances for lodging, light, fuel, washing, and partial board would entail a heavier charge on public funds, whereas the benefits which would accrue to both patients and nurses would be manifold and lasting.

[We shall welcome correspondence on this subject.—Ed., T.H.]

